# Skeletal Muscle NADPH Oxidase Is Increased and Triggers Stretch-Induced Damage in the *mdx* Mouse

**DOI:** 10.1371/journal.pone.0015354

**Published:** 2010-12-20

**Authors:** Nicholas P. Whitehead, Ella W. Yeung, Stanley C. Froehner, David G. Allen

**Affiliations:** 1 Discipline of Physiology, Bosch Institute, The University of Sydney, Sydney, New South Wales, Australia; 2 Department of Physiology & Biophysics, University of Washington, Seattle, Washington, United States of America; 3 Department of Rehabilitation Sciences, Hong Kong Polytechnic University, Hung Hom, Kowloon, Hong Kong, Special Administrative Region, People's Republic of China; McMaster University, Canada

## Abstract

Recent studies have shown that oxidative stress contributes to the pathogenesis of muscle damage in dystrophic (*mdx*) mice. In this study we have investigated the role of NADPH oxidase as a source of the oxidative stress in these mice. The NADPH oxidase subunits gp91^phox^, p67^phox^ and rac 1 were increased 2–3 fold in tibilais anterior muscles from *mdx* mice compared to wild type. Importantly, this increase occurred in 19 day old mice, before the onset of muscle necrosis and inflammation, suggesting that NADPH oxidase is an important source of oxidative stress in *mdx* muscle. In muscles from 9 week old *mdx* mice, gp91^phox^ and p67^phox^ were increased 3–4 fold and NADPH oxidase superoxide production was 2 times greater than wild type. In single fibers from *mdx* muscle NADPH oxidase subunits were all located on or near the sarcolemma, except for p67^phox^,which was expressed in the cytosol. Pharmacological inhibition of NADPH oxidase significantly reduced the intracellular Ca^2+^ rise following stretched contractions in *mdx* single fibers, and also attenuated the loss of muscle force. These results suggest that NADPH oxidase is a major source of reactive oxygen species in dystrophic muscle and its enhanced activity has a stimulatory effect on stretch-induced Ca^2+^ entry, a key mechanism for muscle damage and functional impairment.

## Introduction

Duchenne muscular dystrophy (DMD) is an X-linked, degenerative muscle disease, which affects approximately 1 in 3500 males globally. DMD is caused by the absence of dystrophin, a large (427 kDa) protein connecting the cytoskeleton to a complex of sarcolemmal proteins, which bind to the extracellular matrix. Dystrophin was originally thought to have an important structural and stabilizing role during muscle contractions, which protected muscles from contraction-induced, mechanical damage to the membrane [1]. However, recent evidence from dystrophin-deficient muscles has revealed a more complicated picture, with the abnormal regulation of many ion channels and cell signaling pathways contributing to the disease pathophysiology [2]. In young DMD patients, muscle damage is followed by regeneration but as the disease manifests, regeneration is impeded and muscle fibers are progressively replaced with connective tissue and fatty deposits. Profound muscle weakness results in loss of mobility by about the age of 10–12, and eventually death around age 20–30, due to respiratory and/or cardiac failure.

Reactive oxygen species (ROS) have been implicated in a wide-range of human diseases. Over two decades ago, ROS were postulated to contribute to the pathogenesis of DMD, which led to a number of clinical trials using antioxidants [3]. Overall, these trials were disappointing in terms of clinical benefits. Retrospectively though, these clinical studies were often carried out on patients with advanced muscle degeneration and the antioxidants used were not membrane permeable. This point is highlighted by recent studies on the *mdx* mouse, an animal model of DMD, in which membrane permeable antioxidants administered to young mice ameliorate the progression of damage in both skeletal [4], [5], [6] and cardiac [7] muscle. Therefore, the timing and targeting of the antioxidant are both key factors to consider in designing these drugs as therapeutic strategies for DMD. In recent studies, we have also shown that ROS play an important role in *mdx* muscle damage produced by stretched (eccentric) contractions [6], [8]. Dystrophic muscles are extremely vulnerable to damage from stretched contractions, which are performed during everyday activities, such as walking downhill, when the quadriceps muscle acts as a brake to control the degree of knee flexion against the force of gravity.

An important follow up question to previous studies on oxidative stress is to elucidate the source(s) of excessive ROS production in dystrophic muscles. Given the inflammatory nature of dystrophy, an obvious potential source are ROS-producing cells such as macrophages and neutrophils. However, there is evidence that *mdx* muscles are oxidatively stressed before the onset of observable histological muscle damage and inflammation, which begins at around 3 to 4 weeks of age. For instance, increased expression of endogenous antioxidants, SOD and catalase, as well as lipid peroxidation in pre-necrotic *mdx* mice (up to 20 days of age) have been observed [9]. Similarly, increased levels of oxidized GSH, a measure of cellular ROS reactivity, in muscles of *mdx* mice of the same age, has been shown [10]. These findings imply that the loss of dystrophin initially triggers increased ROS production by a skeletal muscle-specific source, rather than by invading inflammatory cells, which are likely to contribute to oxidative stress during the damage/regeneration phase of the disease.

NADPH oxidase is a multi-protein, enzyme complex, which uses NADPH as a substrate to convert molecular oxygen to reactive oxygen species (ROS), usually superoxide or hydrogen peroxide (H_2_O_2_). It is highly expressed in inflammatory cells, such as neutrophils and macrophages, and is activated during phagocytosis to kill invading pathogens. The phagocyte NADPH oxidase consists of the membrane-bound proteins gp91^phox^, also called NOX2, and p22^phox^. In addition, there are several cytosolic subunits; the organizer subunit, p47^phox^, the activator subunit p67^phox^, the transport subunit p40^phox^, and the small GTP-ases rac1 or rac2, which are required for full activation of the enzyme [11]. This phagocytic NADPH oxidase complex is also present in a many other cell types including all muscle types; smooth, cardiac and skeletal [12]. The only phagocytic NADPH oxidase subunit not detected in skeletal muscle is p40^phox^ [13]. Gene array data has shown significantly increased mRNA for the NADPH oxidase subunits gp91^phox^ [14] and p67^phox^ [15] in *mdx* hindlimb muscles. Moreover, NADPH oxidase activity is increased in *mdx* cardiac muscle [7], [16] and in *mdx* skeletal muscle fibers at rest and subjected due to hypo-osmotic swelling [17]. These results suggest that NADPH oxidase is an important source of ROS in *mdx* muscle, after the initial onset of muscle damage and regeneration. However, to test if NADPH oxidase is a major source of ROS in *mdx* muscle, studies are also needed on pre-necrotic *mdx* mice, younger than 3 weeks of age, to avoid the confounding effects of ROS stimulated by muscle degradation pathways and infiltrating inflammatory cells.

In the current study, we tested the hypothesis that NADPH oxidase is a major source of ROS production in *mdx* muscle, by measuring NADPH oxidase protein levels in pre-necrotic *mdx* mice. We also measured NADPH oxidase enzyme activity and protein expression in young adult *mdx* muscles. Finally, we investigated if NADPH oxidase contributed to stretch-induced muscle damage in *mdx* single fibers. In previous papers we have shown that both the stretch-induced force loss and the rise in intracellular Ca^2+^ are significantly attenuated when stretch-activated channel (SAC) blockers are used [18]. We have also shown that ROS stimulate Ca^2+^ entry through stretch-activated channels in *mdx* fibers, both at rest and following stretched contractions [8]. The data from this previous study also suggested that stretch-activated channels from *mdx* but not wild type muscle are hypersensitive to oxidative modification, either directly or via other ROS-activated signaling proteins such as src kinase. Therefore, in the present study, we investigated whether NADPH oxidase is a source of the stretch-induced ROS, which triggers increased intracellular Ca^2+^ in *mdx* muscle fibers.

## Methods

### Ethics Statement

Wild type (WT) and dystrophin-deficient *mdx* mice, both bred on the C57BL/10ScSnJ background, were purchased from Animal Resources Centre, Perth, Australia and from The Jackson Laboratory, USA. All experimental procedures were approved by the Animal Ethics Committee of the University of Sydney (protocol number: K22/6-2007/2/4625) and the Institutional Animal Care and Use Committee at the University of Washington (protocol number: 3298-02). Mice were euthanized either by I.P injection of 163 mg/kg pentobarbitone sodium (University of Sydney) or by CO_2_ inhalation followed by cervical dislocation (University of Washington).

### Western Blotting

Tibialis anterior (TA) muscles of 18–19 day and 9 week old mice were homogenized (Polytron PT 1200, Kinematica, Littau/Lucerne, Switzerland) in a lysis buffer containing; 50 mM Tris (pH 7.5), 150 mM NaCl, 25 mM EGTA, 25 mM EDTA, 1% Triton X-100, protease inhibitor cocktail, calpain 1 inhibitor and phosphatase inhibitor (Sigma, USA). After 30 min incubation on ice, samples were centrifuged at 15,800 *g* at 4°C and the supernatant was collected. Total protein was measured by the Bradford assay technique (Bio-Rad, Hercules, CA, USA).

TA muscle lysates were immunoblotted as described previously [6]. Membranes were probed using mouse antibodies against rac1 (1∶500) (Cytoskeleton, CO), gp91^phox^ (1∶1000) and p67^phox^ (1∶500) (BD Biosciences, San Jose, CA), and rabbit antibodies against p22^phox^ (1∶500) and p47^phox^ (1∶500) (Santa Cruz, CA). The protein bands were detected with an ECL Plus kit (Amersham Pharmacia Biotech, UK) and visualized with an Alpha Innotech FluoChem SP Imaging System (San Leandro, CA, USA). Densitometry was performed using ImageJ software. Following imaging, membranes were incubated in Ponceau S solution (Sigma, USA), to conform equal loading of protein in each well.

### NADPH-dependent superoxide assay

NADPH oxidase superoxide production was measured with the lucigenin chemiluminescent assay, using similar methods to those previously described [7]. Briefly, TA muscles from WT and *mdx* mice were homogenized in a detergent-free lysis buffer (pH 7.4) containing (mM); 150 NaCl, 50 Tris, 25 EGTA, 25 EDTA, as well as a protease inhibitor cocktail and phosphatase inhibitors. Protein concentrations of each sample were determined by Bradford assay. A total of 2 mg/ml of the protein homogenate, diluted in the same Tris buffer, was used for each experiment. Immediately before the experiment, NADPH (200 µM) and lucigenin (10 µM) were added from stock solutions, so that the final volume was 600 µl. Each sample was incubated at 37°C and the lucigenin-dependent light emission was detected by a photomultiplier-based luminometer. Various drugs were added to block potential sources of ROS; the non-specific NADPH oxidase inhibitor diphenyleneiodonium (DPI, 10 µM); the NOS inhibitor, N-nitro-L-arginine methyl ester hydrochloride (L-NAME, 100 µM); the mitochondrial site I electron transport inhibitor, rotenone (20 µM); and the xanthine oxidase inhibitor, oxypurinol (100 µM).

### NADPH oxidase immunostaining in single fibers

Flexor digitorum brevis (FDB) single fibers from 3 to 8 week old WT and *mdx* mice were isolated based on previous methods [19]. Briefly, FDB muscles were dissected in a solution containing; 138 mM NaCl, 2.7 mM KCl, 1.8 mM CaCl_2_, 1.06 mM MgCl_2_, 12.5 mM HEPES and 5.6 mM glucose. Muscles were then placed in the same solution containing 0.2% collagenase type I and 10% FCS and incubated with 5% CO_2_ in air at 37°C for 90 min. Muscles were placed in culture medium (DMEM and 10% FCS) for 30 min and then single fibers were dissociated by gently triturating with a plastic Pasteur pipette. Isolated fibers were plated in 24 well culture dishes on laminin-coated coverlsips and incubated at 37°C for at least 4 hours to allow for attachment. Single fibers were rinsed with PBS and fixed for 10 min (2% paraformaldehyde). Fibers were the permeabilized and blocked in PBS containing 0.25% saponin, 10% FCS and 1% BSA for 30 min. Primary antibodies, diluted in PBS and 0.1% saponin, were incubated overnight at 4°C. Secondary antibodies (AlexaFluor 488 or 555; Molecular Probes, Invitrogen), diluted 1∶1000 were incubated with 0.1% saponin and DAPI in PBS for 2 hrs at room temperature. Fluorescent images were taken by scanning confocal microscopy (Zeiss LSM 510 Meta), with 40 or 100 x oil immersion objectives, and viewed with Image J software.

### Stretched contractions on *mdx* single fibers

Single muscle fibers from *mdx* mice were loaded with a Ca^2+^ indicator and subjected to stretched contractions, as described previously [18]. Briefly, single FDB muscle fibres from *mdx* mice (8–10 weeks of age) were dissected and attached, via aluminium tendon clips, to a force transducer and length controller. Fibers were perfused with a solution containing (mM); NaCl 121, KCl 5, CaCl_2_ 1.8, MgCl_2_ 0.5, NaH_2_P0_4_ 0.4, NaHCO_3_ 24 and glucose 5.5. The solution was maintained at room temperature and constantly bubbled with 95% O_2_–5% CO_2_ (pH 7.4). Fibers were loaded with 10 µM Fluo4-AM (Molecular Probes) to measure the resting intracellular Ca^2+^ concentration ([Ca^2+^]_i_). Experiments were carried out on non-treated fibers (control) and fibers treated for 15 min with the NADPH oxidase inhibitor DPI (1 µM). Confocal microscopy was used to measure changes in [Ca^2+^]_i_ before and after 10 stretched contractions, using our previous protocol [18]. Isometric force was also measured before and 10 min after the stretched contractions.

### Statistics

All results are presented as the mean ± SEM. Statistical differences between groups (WT or *mdx*, control or treated) were determined using the Student's unpaired *t-*test. The level of statistical significance was set at *P*<0.05.

## Results

### NADPH oxidase proteins are increased in 9 week old *mdx* muscle

**Figure 1:**
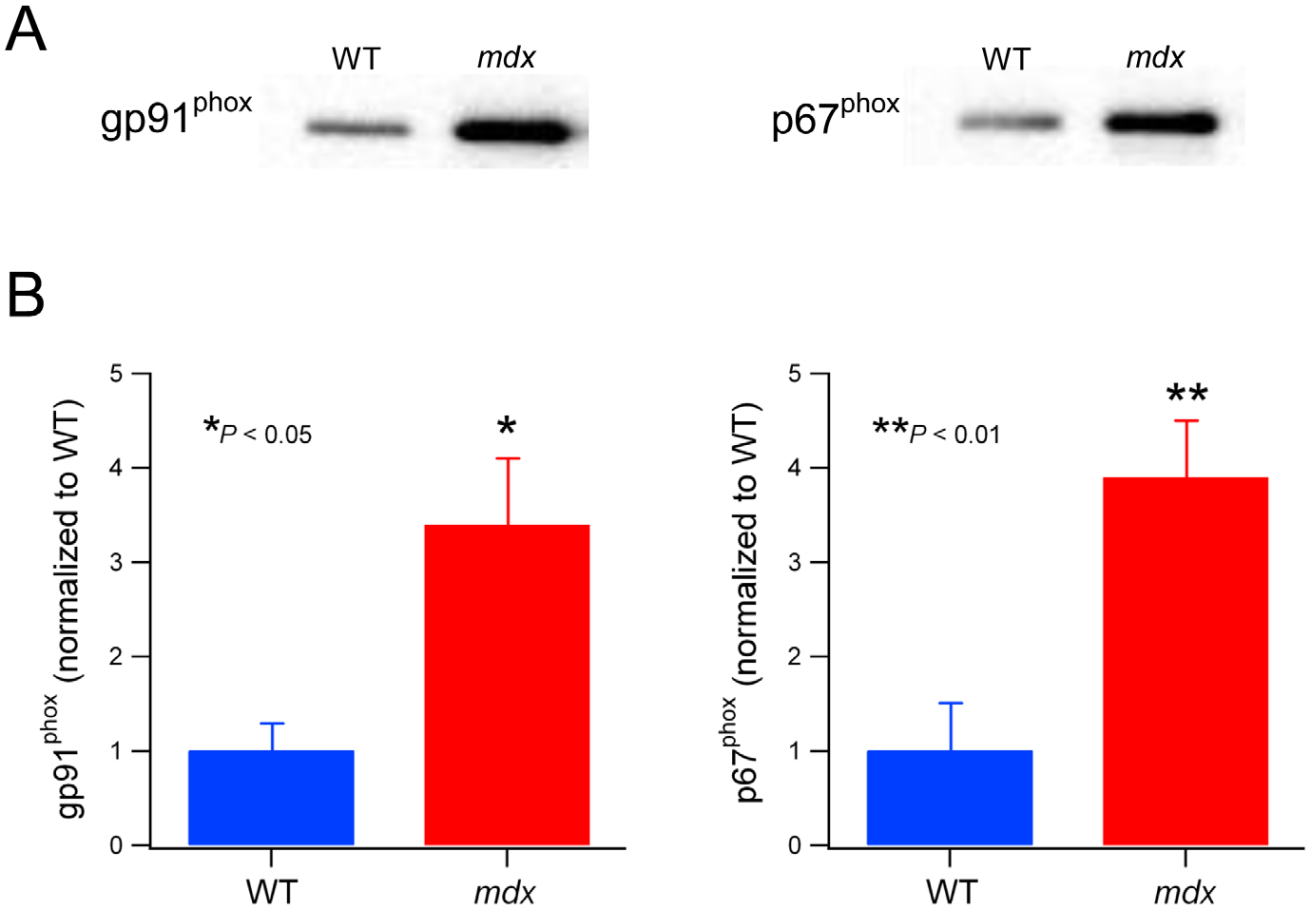
NADPH oxidase subunits are increased in 9 week old *mdx* mice. A. Representative western blots of gp91^phox^ and p67^phox^ from 9 week old WT and *mdx* TA muscles. B. Pooled results of densitometry analysis for gp91^phox^ and p67^phox^ from WT (n = 4) and *mdx* (n = 8) muscles.

The NADPH oxidase catalytic subunit gp91^phox^ is increased in 4–8 week old *mdx* skeletal muscles [17]. In the current study, we found a similar magnitude increase (3–4 fold) in TA muscle homogenates from 9 week *mdx* mice, compared to WT (Figure 1). In addition, at this age, we also measured the expression levels of the key activator subunit, p67^phox^. Interestingly, we found that this protein also increased by 3–4 fold in *mdx* muscle (see Figure 1). Both of these proteins were significantly greater for *mdx* compared to WT (gp91^phox^, *P*<0.05; p67^phox^, *P*<0.01).

### NADPH oxidase-dependent superoxide production is increased in *mdx* muscle

**Figure 2:**
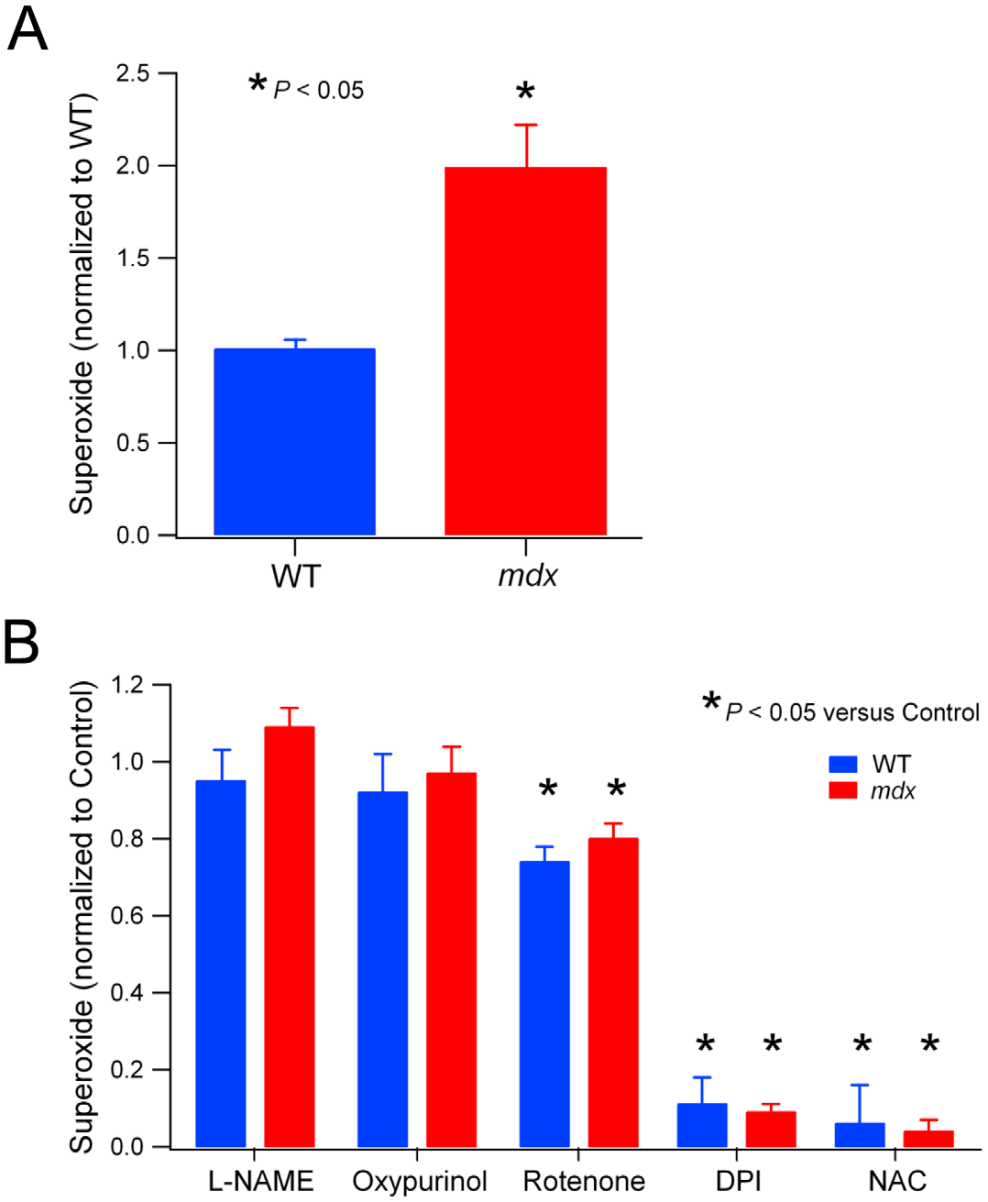
NADPH oxidase superoxide production is increased in *mdx* muscle. A. Lucigenin chemiluminescent assay was used to measure NADPH-dependent superoxide production from muscle homogenates of WT and *mdx* mice (see Methods for details). Values from each experiment were pooled for WT and *mdx* muscles (n = 5 in both groups). B. Inhibitors of various sources of ROS were added to the assay and superoxide production was compared to the control level (no inhibitor). Pooled values for WT and *mdx* mice.

Given the increased protein expression of NADPH oxidase in *mdx* muscle, we then investigated whether this would result in enhanced NADPH oxidase activity. Previously, our lab showed that NADPH oxidase activity was increased in heart homogenates from *mdx* mice [7]. Therefore, we used the same procedure to investigate whether NADPH oxidase ROS production was increased in *mdx* skeletal muscle compared to WT. NADPH-dependent ROS (superoxide) production was measured in TA muscle homogenates from 9 week old WT and *mdx* mice. We used a well-established technique, the lucigenin chemiluminescent superoxide assay, using NADPH as the substrate (see Methods for details). Superoxide production was significantly increased (*P*<0.05) by 2 fold in *mdx* muscle compared to WT (Figure 2A). To establish that NADPH oxidase was the primary source of the increased superoxide production in *mdx* muscles, we used inhibitors of NADPH oxidase and the other main cellular ROS sources (Figure 2B). For both WT and *mdx* muscles, L-NAME and oxypurinol had no effect, while rotenone reduced superoxide by about 20% (*P*<0.05). However, the NADPH oxidase inhibitor DPI prevented more than 90% of the superoxide production (*P*<0.05), indicating that NADPH oxidase was the main source of the ROS in this assay. Like DPI, the antioxidant NAC, used as a positive control, almost completely prevented superoxide production (*P*<0.01).

### NADPH oxidase subunits are differentially increased in 18–19 day old (pre-necrotic) *mdx* mice

**Figure 3:**
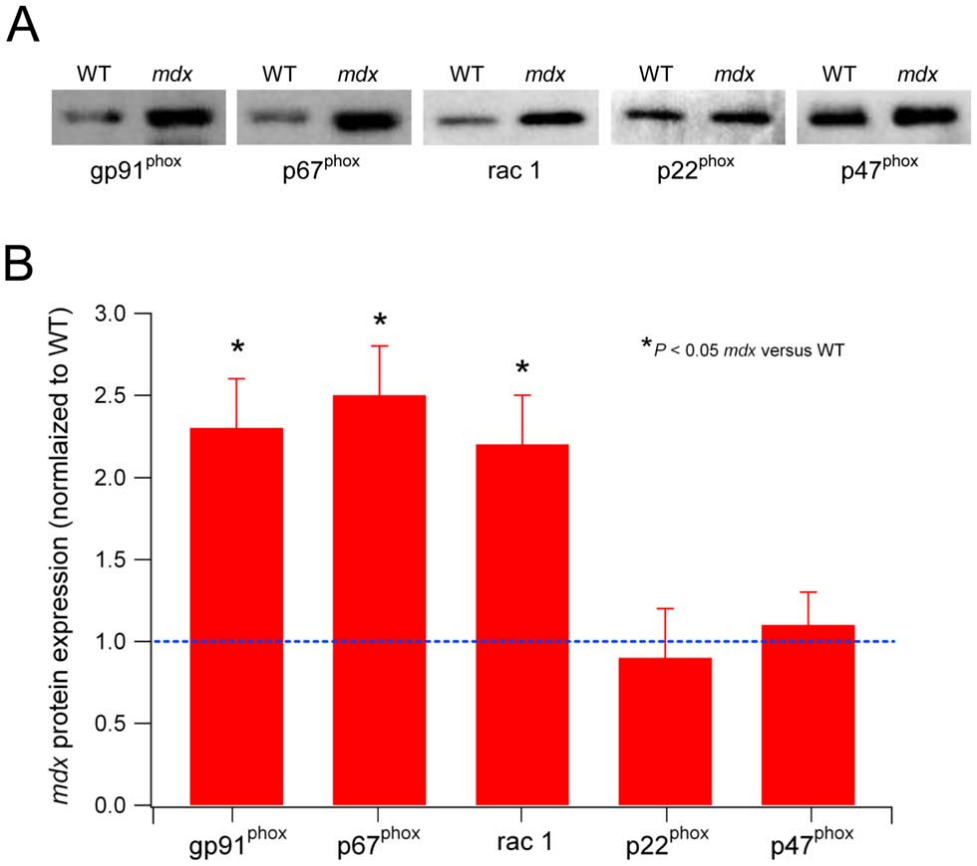
NADPH oxidase subunits are increased in 18–19 day old (pre-necrotic) *mdx* mice. A. Representative western blots of all NADPH oxidase subunits from TA muscles of 18–19 day old WT and *mdx* mice. B. Pooled results from densitometry for the subunits showing *mdx* values normalized to WT (represented by the blue dotted line). The proteins gp91^phox^, p67^phox^ and rac1 were all significantly increased in *mdx* muscle compared to WT, whereas p22^phox^ and p47^phox^ were not different (n = 4 samples for each group).

Between the ages of about 4 and 8 weeks, *mdx* muscles undergo extensive cycles of necrosis, inflammation and regeneration. Since inflammatory cells such as macrophages and neutrophils express NADPH oxidase, we wanted to test whether NADPH oxidase protein expression was increased in 18–19 day old, pre-necrotic, *mdx* muscles. This would determine whether the increased NADPH oxidase expression in *mdx* muscles was produced by skeletal muscle fibers, as a consequence of the lack of dystrophin, and not from inflammatory cells. In these experiments, we measured protein expression levels of all the NADPH oxidase subunits. As shown in Figure 3, three subunits, gp91^phox^, p67^phox^ and rac1 were all significantly increased by 2–3 fold in *mdx* TA muscles compared to WT (*P*<0.05). The levels of the other two subunits, p22^phox^ and p47^phox^ were not significantly different between *mdx* and WT.

**Figure 4:**
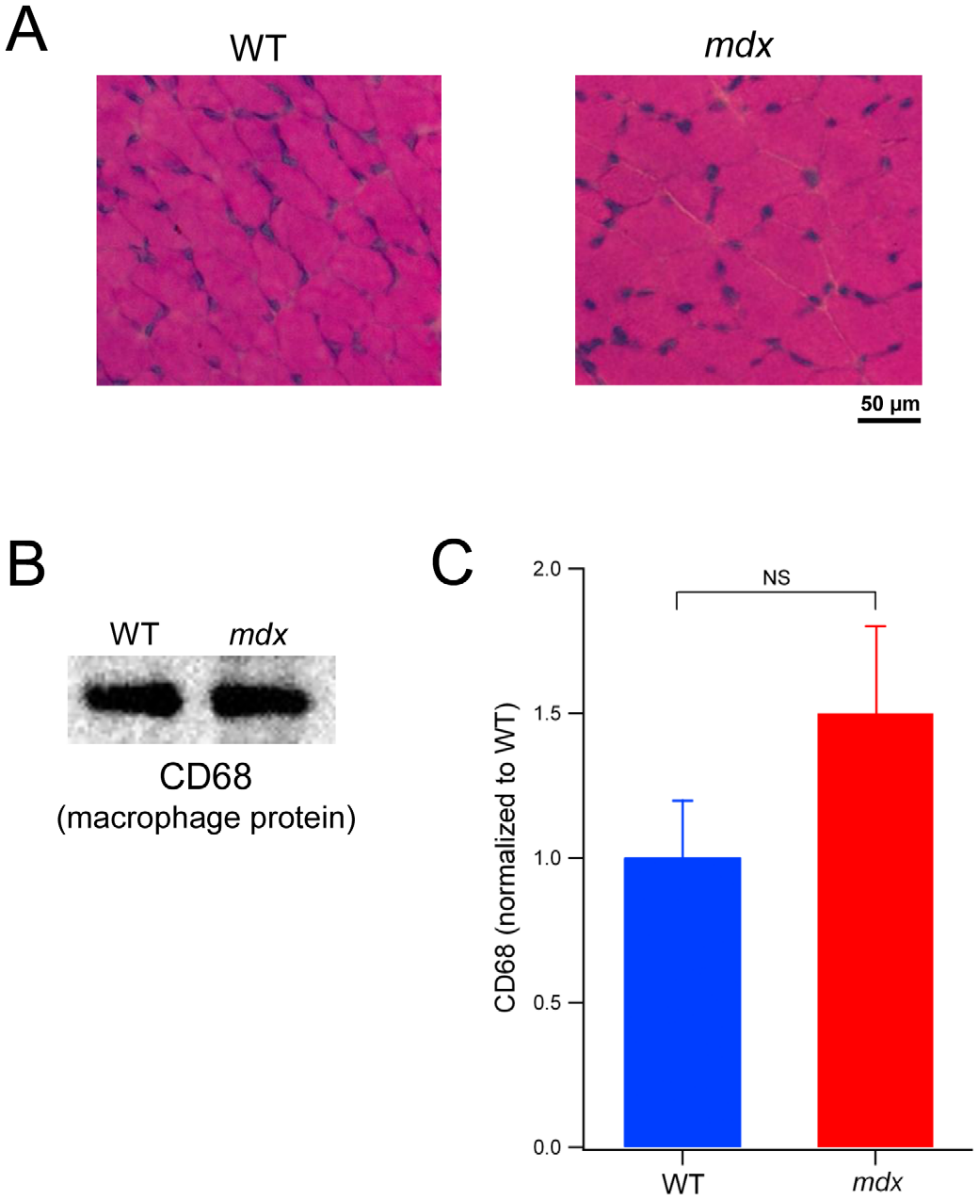
Muscles of 18–19 day old *mdx* mice show no evidence of inflammatory cells. A. Representative images of TA cross-sections from 19 day old WT and *mdx* mice stained with H&E. Note the normal histological appearance of the *mdx* muscle, which is devoid of necrotic fibers and infiltrating inflammatory cells. B. Sample western blot showing the macrophage protein, CD68, from TA homogenates of 18–19 day old WT and *mdx* mice. C. Pooled data of CD68 expression from western blots of 18–19 day old mice, showing no significant difference (NS) between WT and *mdx* muscles.

In order to confirm that the higher expression of NADPH oxidase was not derived from increased numbers of inflammatory cells, we firstly examined histological sections, by H&E staining. We found no evidence of inflammatory cell infiltration or myofiber necrosis in 18–19 day old *mdx* mice (Figure 4A). In addition, we also showed by immunoblotting that the macrophage protein CD68 was not significantly different between *mdx* and wild type mice at this age (Figure 4B and C).

### Localization of NADPH oxidase proteins in single muscle fibers

**Figure 5:**
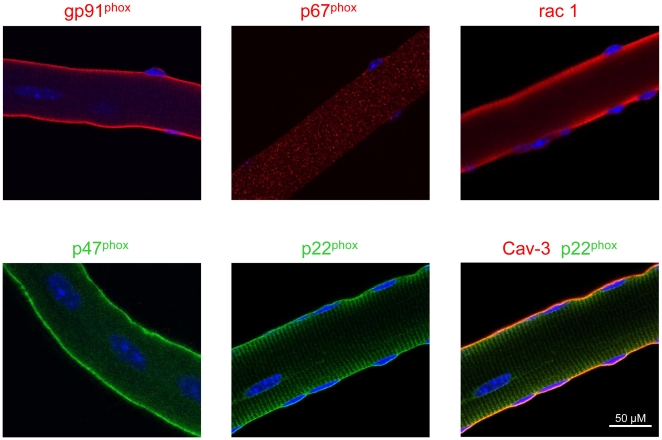
NADPH oxidase immunostaining of single fibers from *mdx* mice. Isolated *mdx* muscle fibers from the FDB muscle were immunostained using antibodies against the various NADPH oxidase subunits. Nuclei are stained by DAPI (blue). In the bottom right panel, p22^phox^ (green) was co-immunostained with caveolin-3 (red) to demonstrate sarcolemmal localization (yellow).

To determine the cellular localization of NADPH oxidase subunits we carried out immunocytochemistry on isolated single muscle fibers. Mice ranging in age from 3 to 9 weeks of aged were used and, in general, there was no observable difference in the localization of the various subunits as a function of age. Furthermore, the localization was similar between *mdx* and WT fibers. All of the subunits, with the exception of p67^phox^, were localized on or near the sarcolemma (Figure 5). A striated pattern of staining for gp91^phox^ and particularly p22^phox^ is probably due to *t*-tubule expression [20]. In addition to sarcolemmal localization, p47^phox^ is diffusely found in the cytoplasm, while p67^phox^ is only located in the cytoplasm in resting cells. In order to demonstrate sarcolemmal localization, p22^phox^ (green) was co-labeled with caveolin-3 (red), the muscle-specific caveolin isoform, found in sarcolemmal caveolae. As shown in Figure 5, there was good co-localization between the two proteins, characterized in the merged image by the yellow staining along the sarcolemma.

### Stretch-induced Ca^2+^ influx in *mdx* fibers is reduced by an NADPH oxidase inhibitor

**Figure 6:**
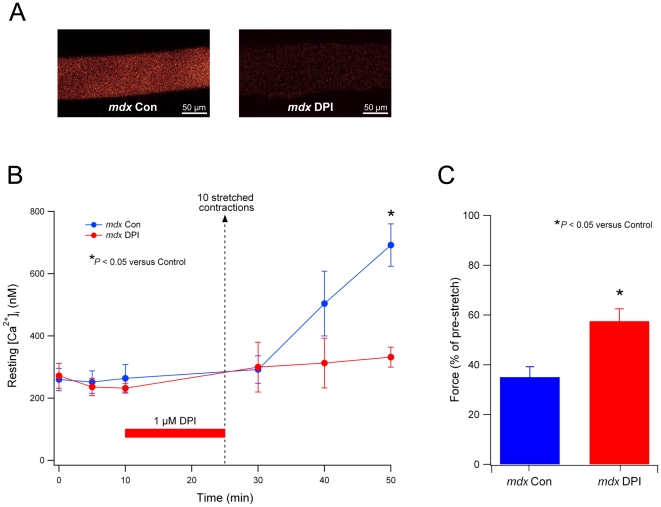
NADPH oxidase inhibition reduces stretch-induced Ca^2+^ influx and force loss in *mdx* fibers. A. Fluorescent confocal images showing intracellular Ca^2+^ (Fluo-4) for *mdx* single fibers taken 25 min after a series of 10 stretched contractions. Fibers were either untreated controls (*mdx* Con) or treated with the NADH oxidase inhibitor DPI (*mdx* DPI). B. Pooled data of the resting intracellular Ca^2+^ concentration ([Ca^2+^]_i_) as measured before and after 10 stretched contractions (dotted line with arrow). Values are shown for control fibers (n = 9; blue) and fibers treated with 1 µM DPI (n = 5; red) for 15 min before the stretched contractions. C. Pooled values showing the significantly greater muscle force after the stretched contractions for DPI- treated fibers (n = 5; red) compared to control fibers (n = 9; blue). Note that pre-stretch force values are represented by 100%.

We have previously shown that the stretch-induced Ca^2+^ influx in *mdx* fibers occurs through stretch-activated channels [18]. Recently, we showed that stretch-induced ROS mediate this effect, since the Ca^2+^ influx could be prevented with an antioxidant [8]. In the current study, we extended these findings by determining whether NADPH oxidase was the source of the stretch-induced ROS, which triggers the opening of the SACs and the Ca^2+^ influx. In these experiments, we used only *mdx* fibers, since WT fibers show only a very small rise in resting intracellular Ca^2+^ following our stretched contraction protocol [18]. Single *mdx* fibers were incubated with or without the NADPH oxidase inhibitor DPI (1 µM) for 15 min before a series of 10 stretched contractions. The intracellular Ca^2+^ indicator Fluo-4 was used to measure changes in [Ca^2+^]_i_ before and after the stretched contractions, using confocal microscopy. As shown previously [8], Ca^2+^ levels increased progressively for 25 min after the stretched contractions in *mdx* control fibers (Figure 6A and B). DPI greatly reduced the rise in Ca^2+^ after the stretched contractions, significant at 25 min compared to control fibers (*P*<0.05). As shown in Figure 6C, the reduction in muscle force after the stretched contractions was also significantly inhibited by DPI (*P*<0.05) by about 20%, which is comparable to the effect provided by SAC blockers [18] and the antioxidant Tiron [8].

## Discussion

Oxidative stress is a hallmark feature of many pathological human conditions, including DMD [21]. In terms of understanding disease mechanisms and designing therapeutic strategies for muscular dystrophy, it is important to discern if this overproduction of ROS is a primary cause or secondary effect of the disease progression. Therefore, identifying the source(s) of the excessive ROS is necessary. In recent years, NADPH oxidase has emerged as an important source of ROS for normal physiological functions. However, enhanced activity is associated with pathological conditions affecting a wide range of tissues and organs, including the heart, kidney, lung and vasculature [12]. In skeletal muscle, relatively little is known about the role and regulation of NADPH oxidase. Recent work from us and others, using various antioxidants, has shown that ROS is a cause of both the chronic muscle damage in *mdx* mice and the acute damage from stretched contractions [4], [6], [8], [22]. In the current study, we hypothesized that NADPH oxidase was a major source of the enhanced ROS production in *mdx* skeletal muscle. Recent studies showed that protein expression of gp91^phox^ was increased 3 fold in muscles from 4–8 week old *mdx* mice compared to wild type [17]. We confirmed this result in 9 week old *mdx* mice and additionally showed that the activator subunit, p67^phox^ was increased by a similar amount. In addition, we found a two-fold increase in NADPH-dependent superoxide production from *mdx* muscle homogenates, which was almost completely prevented by the non-specific NADPH oxidase inhibitor DPI but not by blockers of NOS or xanthine oxidase, other possible sources of ROS. The mitochondrial complex I inhibitor, rotenone, provided a small but significant attenuation of superoxide, however this difference was the same for WT and *mdx* muscles. Therefore, this data suggests that NADPH oxidase ROS generation is greatly enhanced in *mdx* skeletal muscle, consistent with findings in *mdx* cardiac muscle [7].

Dystrophic muscles are characterized by cycles of myofiber damage, necrosis, inflammation and finally regeneration or fibrosis. In *mdx* mouse hind limb muscles, such as TA, EDL and soleus, this process is most prominent between about 4–9 weeks of age, during which time more than 50% of the muscle undergoes damage and regeneration, as evidenced by the number of fibers with central nuclei. During this period, infiltrating inflammatory cells are a key feature of the disease and can both exacerbate muscle damage as well as promote regeneration [23]. Upon activation, macrophages and neutrophils utilize NADPH oxidase to produce superoxide, which reacts to form more cytotoxic ROS that can damage neighboring cells. In skeletal muscle, ROS production by neutrophil NADPH oxidase has been shown to cause significant muscle damage following hindlimb unloading, which was attenuated in gp91^phox^ null mice [24]. Thus, in order to show that skeletal muscle, and not phagocyte, NADPH oxidase is a major cause of oxidative stress in dystrophic muscle, we measured protein expression levels in young (18–19 day old) *mdx* mice before the onset of muscle damage and inflammatory cell infiltration and activation. Importantly, in young *mdx* muscle, we found a significant, 2–3 fold, increase in three NADPH oxidase subunits; gp91^phox^, p67^phox^ and rac1. Expression of the other two subunits, p47^phox^ and p22^phox^ was not different between *mdx* and WT. In *mdx* muscles we found no histological evidence of muscle necrosis, or inflammatory cell infiltration, and expression of the macrophage protein, CD68, was not different between *mdx* and WT muscles. Normal muscles contain resident, non-activated, macrophages [25], which probably explains the detection of CD68 in WT and *mdx* muscles. Therefore, given the immunofluorescent detection of all NADPH oxidase subunits in single myofibers (see Figure 5), we postulate that the increased NADPH oxidase subunit levels in *mdx* muscles is most likely due to enhanced expression by skeletal muscle fibers themselves. As mentioned previously, there is evidence of oxidative stress in pre-necrotic *mdx* muscles [9], [10]. While other sources of ROS, such as mitochondria and xanthine oxidase might also contribute to the enhanced ROS production, our data suggests that NADPH oxidase is a major source of oxidative stress in dystrophin-deficient muscle, a conclusion also reached by Shkryl *et al.*,(2009) [17] (see below).

An interesting finding was the differential changes in the expression of the various NADPH oxidase subunits in *mdx* muscle, with gp91^phox^, p67^phox^ and rac1 increased, and p22^phox^ and p47^phox^ the same as WT. This could be explained by different transcriptional regulation of the genes encoding these proteins. In support of our findings, both gp91^phox^ [14] and p67^phox^ [15] mRNA levels are greatly increased in *mdx* skeletal muscles. Transcriptional regulation of these subunits in *mdx* muscle is possibly mediated by the transcription factor NF-κB regulates, which is known to regulate NADPH oxidase gene transcription in phagocytes [26]. The DNA binding activity of NF-κB is greatly increased in the diaphragm of pre-necrotic 15 and 18 day old *mdx* mice [27], which supports the interpretation that NF-κB activity is involved in the upregulation of these subunits. Moreover, the same study showed that the ROS scavenger NAC reduced NF-κB activity in *mdx* muscle. Thus, a positive feedback loop whereby NF-κB increases NADPH oxidase expression and the resulting ROS production further stimulates NF-κB nuclear translocation and transcriptional activity may occur. Consistent with this idea, we have recently shown that chronic treatment of *mdx* mice with NAC, from 3 weeks of age, attenuates muscle damage and significantly reduces nuclear localization of NF-κB [6]. Currently, it is not clear why the other NADPH oxidase proteins, p22^phox^ and p47^phox^ did not also increase in *mdx* muscle. In normal mouse skeletal muscle, p22^phox^ is the most abundant and p67^phox^ the least abundant subunit, at least at the mRNA level [28]. Thus, assuming a stoichiometry of 1∶1 for the NADPH oxidase subunits [29], it is necessary for the least expressed subunits to be upregulated in order to form a greater number of active NADPH oxidase complexes. The three subunits that increased in the current experiments are all critical for full activation of the enzyme: p67^phox^ and rac1 bind to each other and gp91^phox^ to stimulate catalytic activity of the enzyme [12]. On the other hand, while p47^phox^ is considered essential for activation in phagocytes and other cell types following agonist stimulation, it is not required for basal activity in smooth muscle cells [30]. Future experiments are required to determine the regulation and role of each NADPH oxidase subunit in both normal and dystrophic skeletal muscle.

We have previously shown in *mdx* fibers that the stretch-induced rise in cytosolic Ca^2+^ is mediated by SAC [18]. Moreover, blocking SAC in *mdx* muscles also significantly attenuated the force drop following stretched contractions and reduced membrane permeability [18], [31]. More recently, we found that these effects of SAC blockers could be replicated by antioxidants [6], [8], and in the current study, by the NADPH oxidase inhibitor DPI (see Figure 6). Taken together, these results imply that stretch of *mdx* muscle produces ROS, by activation of NADPH oxidase, which subsequently triggers Ca^2+^ entry through SAC leading to muscle damage and contractile dysfunction. Our interpretation is consistent with recent findings [17]. Using a different model of stretch, hypo-osmotic swelling, Shkryl *et al.*, (2009) reported that NADPH oxidase triggered Ca^2+^ sparks in *mdx* fibers. They postulated that the Ca^2+^, triggered by NADPH oxidase-derived ROS, was later sequestered by mitochondria, which then generated additional ROS. Interestingly, basal ROS production from NADPH oxidase, but not mitochondria, was greater in *mdx* fibers compared to WT. Again, these findings suggest that stretch-induced NADPH oxidase ROS production precedes, and subsequently stimulates mitochondrial ROS production via Ca^2+^ uptake. Consistent with this idea, there is recent evidence that in 5 week old *mdx* mice, during the extensive muscle damage phase, mitochondria also contribute to ROS production [32].

Currently, the mechanism by which NADPH oxidase-dependent ROS activates SAC remains unclear. One possibility is that ROS activates SAC directly, via redox-sensitive sulfhydryl groups, or indirectly, by ROS-mediated signaling pathways. We have recently shown that TRPC1, a candidate for the overactive SAC in *mdx* muscle [33], was activated via ROS sensitive src kinase [8]. Furthermore, TRPC1 and src kinase were increased in *mdx* muscle, and src kinase inhibition significantly improved muscle force following stretched contractions in *mdx* EDL muscle [8]. Src kinase phosphorylates p47^phox^ to initiate its translocation and organization of the other NADPH oxidase cytosolic subunits to the membrane-bound complex [34]. Thus, increased expression and activation of src kinase might be involved in the enhanced stretch-induced ROS production of NADPH oxidase and the associated Ca^2+^ influx. TRPV2 is another potential candidate for the SAC in *mdx* muscle [35]. A final point of consideration is that both TRPC1 [8] and src kinase [36] bind to Caveolin-3, which is upregulated in *mdx* muscles [37]. Here, we have shown that p22^phox^ co-localized with caveolin-3 in single fibers (see Figure 5). We also have preliminary evidence that NADPH oxidase subunits are found in both caveolae and non-caveolae fractions in *mdx* and WT muscles (unpublished observations). Depending on the cell type and species, caveolae or lipid rafts can regulate activation [38] or inhibition [39] of NADPH oxidase. Therefore, in future experiments it will be important to elucidate whether caveolin-3 and caveolae play a role in the regulation of NADPH oxidase activity in *mdx* muscles.

In summary, this study has identified NADPH oxidase as a major source of ROS production in *mdx* muscle, which is upregulated during the period of oxidative stress that precedes muscle damage and inflammation. The other key finding is that NADPH oxidase is involved in the activation of a ROS-sensitive SAC in dystrophic muscle, triggered by stretched contractions. Inhibition of NADPH oxidase prevented the influx of Ca^2+^ into the muscle and provided protection against the force deficit. Therefore, investigating the regulation of NADPH oxidase expression and activity will be important to gain new insight into the mechanisms of damage in dystrophic muscle and to potentially develop new therapeutic approaches for DMD.
